# The effects of prenatal and early postnatal tocotrienol-rich fraction supplementation on cognitive function development in male offspring rats

**DOI:** 10.1186/1471-2202-14-77

**Published:** 2013-07-31

**Authors:** Gowri Nagapan, Yong Meng Goh, Intan Shameha Abdul Razak, Kalanithi Nesaretnam, Mahdi Ebrahimi

**Affiliations:** 1 Malaysian Palm Oil Board, 6 Persiaran Institusi, Bandar Baru Bangi, 43000 Kajang, Selangor, Malaysia; 2 Department of Veterinary Preclinical Sciences, Faculty of Veterinary Medicine, Universiti Putra Malaysia, 43400 UPM Serdang, Selangor, Malaysia; 3 Institute for Tropical Agriculture, Universiti Putra Malaysia, 43400 UPM Serdang, Selangor, Malaysia

**Keywords:** Tocotrienol-rich fraction, Cognitive function, Spatial learning, Brain

## Abstract

**Background:**

Recent findings suggest that the intake of specific nutrients during the critical period in early life influence cognitive and behavioural development profoundly. Antioxidants such as vitamin E have been postulated to be pivotal in this process, as vitamin E is able to protect the growing brain from oxidative stress. Currently tocotrienols are gaining much attention due to their potent antioxidant and neuroprotective properties. It is thus compelling to look at the effects of prenatal and early postnatal tocotrienols supplementation, on cognition and behavioural development among offsprings of individual supplemented with tocotrienols. Therefore, this study is aimed to investigate potential prenatal and early postnatal influence of Tocotrienol-Rich Fraction (TRF) supplementation on cognitive function development in male offspring rats. Eight-week-old adult female Sprague Dawley (SD) rats were randomly assigned into five groups of two animals each. The animals were fed either with the base diet as control (CTRL), base diet plus vehicle (VHCL), base diet plus docosahexanoic acid (DHA), base diet plus Tocotrienol-Rich fraction (TRF), and base diet plus both docosahexaenoic acid, and tocotrienol rich fraction (DTRF) diets for 2 weeks prior to mating. The females (F0 generation) were maintained on their respective treatment diets throughout the gestation and lactation periods. Pups (F1 generation) derived from these dams were raised with their dams from birth till four weeks post natal. The male pups were weaned at 8 weeks postnatal, after which they were grouped into five groups of 10 animals each, and fed with the same diets as their dams for another eight weeks. Learning and behavioural experiments were conducted only in male off-spring rats using the Morris water maze.Eight-week-old adult female Sprague Dawley (SD) rats were randomly assigned into five groups of two animals each. The animals were fed either with the base diet as control (CTRL), base diet plus vehicle (VHCL), base diet plus docosahexanoic acid (DHA), base diet plus Tocotrienol-Rich fraction (TRF), and base diet plus both docosahexaenoic acid, and tocotrienol rich fraction (DTRF) diets for 2 weeks prior to mating. The females (F0 generation) were maintained on their respective treatment diets throughout the gestation and lactation periods. Pups (F1 generation) derived from these dams were raised with their dams from birth till four weeks post natal. The male pups were weaned at 8 weeks postnatal, after which they were grouped into five groups of 10 animals each, and fed with the same diets as their dams for another eight weeks. Learning and behavioural experiments were conducted only in male off-spring rats using the Morris water maze.

**Results:**

Results showed that prenatal and postnatal TRF supplementation increased the brain (4–6 fold increase) and plasma α-tocotrienol (0.8 fold increase) levels in male off-springs. There is also notably better cognitive performance based on the Morris water maze test among these male off-springs.

**Conclusion:**

Based on these results, it is concluded that prenatal and postnatal TRF supplementation improved cognitive function development in male progeny rats.

## Background

Vitamin E is a lipid soluble natural antioxidant. Tocopherols and tocotrienols are the two major families that made up vitamin E. The tocotrienols are the major form of vitamin E found in palm oil, at a typical ratio of 30% tocopherols to 70% tocotrienols. Tocotrienols are similar to tocopherols except that they have an isoprenoid tail with three unsaturation points instead of a saturated phytyl tail. The presence of this unsaturated side chain allows tocotrienols to penetrate into tissues with saturated fatty layers in the cell membrane of brain and liver efficiently [1]. This enables tocotrienols to have far reaching and efficient free radical scavenging properties compared to tocopherols. It is therefore not surprising that the accumulation of tocotrienols in tissues is associated with a range of health benefits. Studies have shown that apart from their potent antioxidant properties [2], tocotrienols are cardioprotective [3], hypocholesterolemic [4], anti-cancerous [5] and neuroprotective [6]. Apart from its antioxidant properties, the latter properties are remotely associated with tocopherols, and thus unique to tocotrienols even though both are important members of the vitamin E family. Tocotrienols are also a nutrient and have recently been certified as GRAS (generally regarded as safe) by the US FDA GRN 307 as of April 2010 [7].

Cognition refers to the mental processes that are involved in memory and learning [8]. Various factors such as nutrition, environment and genetics have strong influence on memory and learning. Nutrition affects cognition and mental health as the brain structure and function are dependent on nutritional inputs [9]. Various dietary factors such n-3 fatty acids, antioxidants, vitamins, minerals, curcumins and flavonoids among others have been identified to have beneficial effects on cognition [10]. These dietary factors affect multiple brain processes involving neurotransmitter pathways, synaptic transmission, membrane fluidity and signal transduction pathways that are associated with synaptic plasticity [10]. In contrast, diets rich in saturated and trans fats are known to affect cognition adversely. This is typically attributed to the role of trans and saturated fats in reducing the synaptic plasticity mediated by the hippocampal brain-derived neurotrophic factor (BDNF) [11].

The brain develops rapidly during the last trimester of fetal life (prenatal) and within the first two years (postnatal) of childhood in humans [12]. During this period, the intake of n-3 and n-6 long chain polyunsaturated fatty acids (LCPUFAs), particularly docosahexanoic acid (DHA), eicosapentaenoic acid (EPA) and arachidonic acid (AA), have been proven to be beneficial for the development of sensory, cognitive and neuromotor systems in human and animal [13]. Many other nutrients such as antioxidant compounds may also fill this role as they are able to protect the vulnerable brain cells from lipid peroxidation [14]. An optimal supply of antioxidants such as vitamin E is thought to be beneficial to the cognitive development in infants. Indeed, optimal supplementation of a specific nutrient during early life could influence or ‘program’ long-term cognitive development, as well as development of major diseases well into adulthood [15].

An accumulation of tocotrienols in the brain is needed to protect the vulnerable neurons from oxidative stress, and to enhance the existing neuronal function as well as neuronal remodelling. Long-term oral supplementation was reported to be effective in delivering tocotrienols to vital organs via tocopherol transfer protein (TTP) independent delivery systems [16]. Delivery is also observed to be more pronounced in the fetal brain when pregnant rats are fed with tocotrienols [17]. Previous animal studies on the effects of tocotrienols on cognition are mostly focused on the role of tocotrienols in improving or preventing cognitive impairments associated with diabetes [18], alcoholism [19], oxidative stress [20] and aging [21]. Apart from the positive health benefits observed during adulthood, we postulated that prenatal and early postnatal tocotrienol supplementation could elicit some degree of protective effects against lipid peroxidative damage in the fetal brain. This would therefore resulted in brain development favorable to the development of healthy mental cognitive functions. Thus, it is the aim of this study to investigate the potential prenatal and early postnatal effects of TRF supplementation on cognitive function development among male progenies, born from dams supplemented with TRF.

## Results

### Plasma vitamin E content

**Figure 1:**
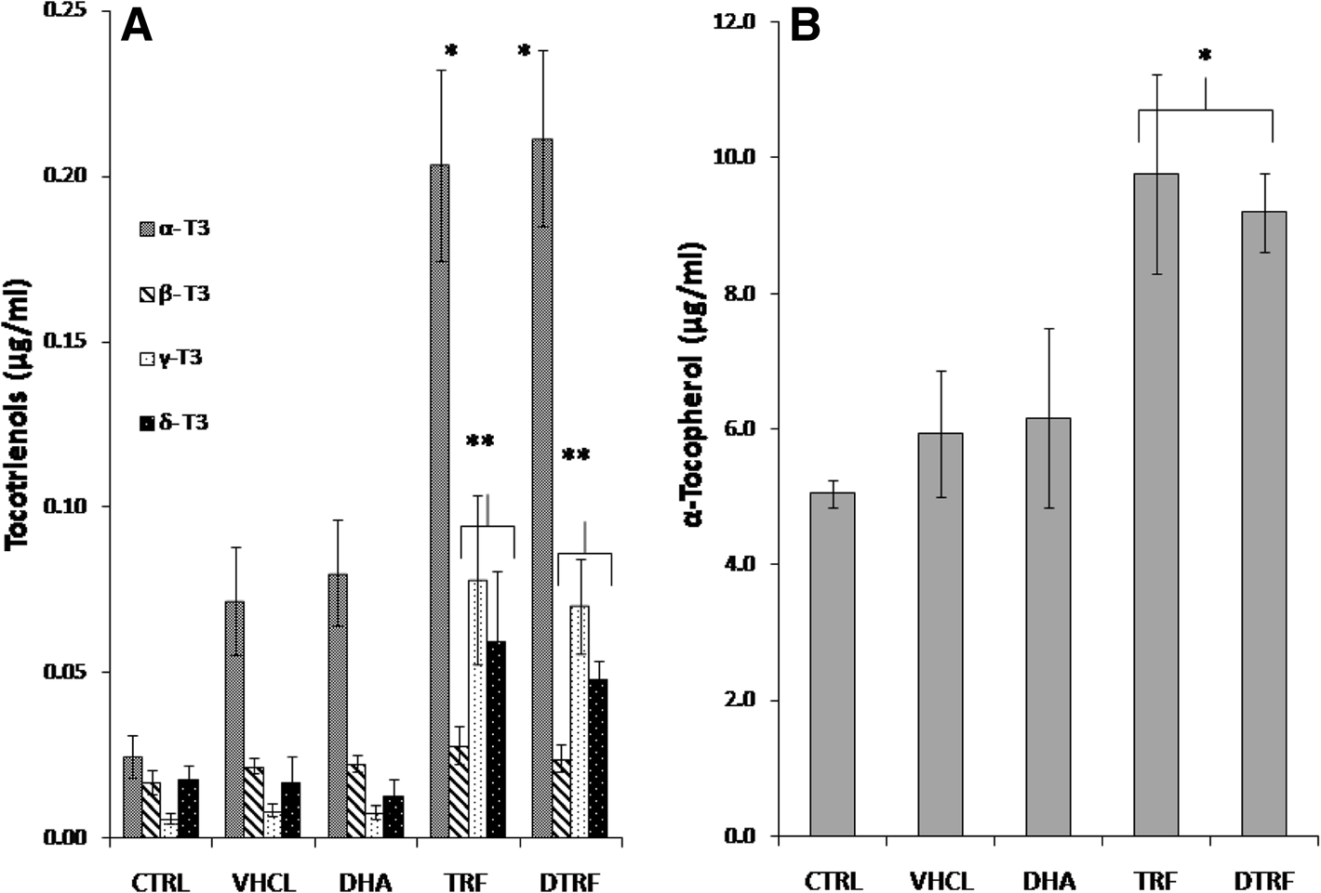
**Vitamin E levels in the plasman of male offspring rats accross treatment groups. A)** Tocotrienol **B)** α-tocopherol. Values are means ± SD, n = 10. Significantly different from the control group, *P ≤ 0.001 and **P ≤ 0.05.

The plasma tocopherol and tocotrienol levels in the male offsprings reflected their respective dietary supplementation levels (Figure 1). α-Tocotrienol and α-tocopherol were the major vitamin E fraction found in plasma followed by γ- and δ-tocotrienol. However, the tocotrienol levels were very much lower than the tocopherol levels in plasma. The levels of α-, γ-and δ-tocotrienol were found to have increased among the TRF and DTRF supplemented animals compared to the control animals. It was also noted that the level of β-tocotrienol fraction was negligible and was no different among the TRF and DTRF supplemented rats. Conversely, the α-tocopherol level was elevated in TRF and DTRF supplemented rats. The levels of other tocopherol fractions were mostly negligible.

### Vitamin E content in the brain

**Figure 2:**
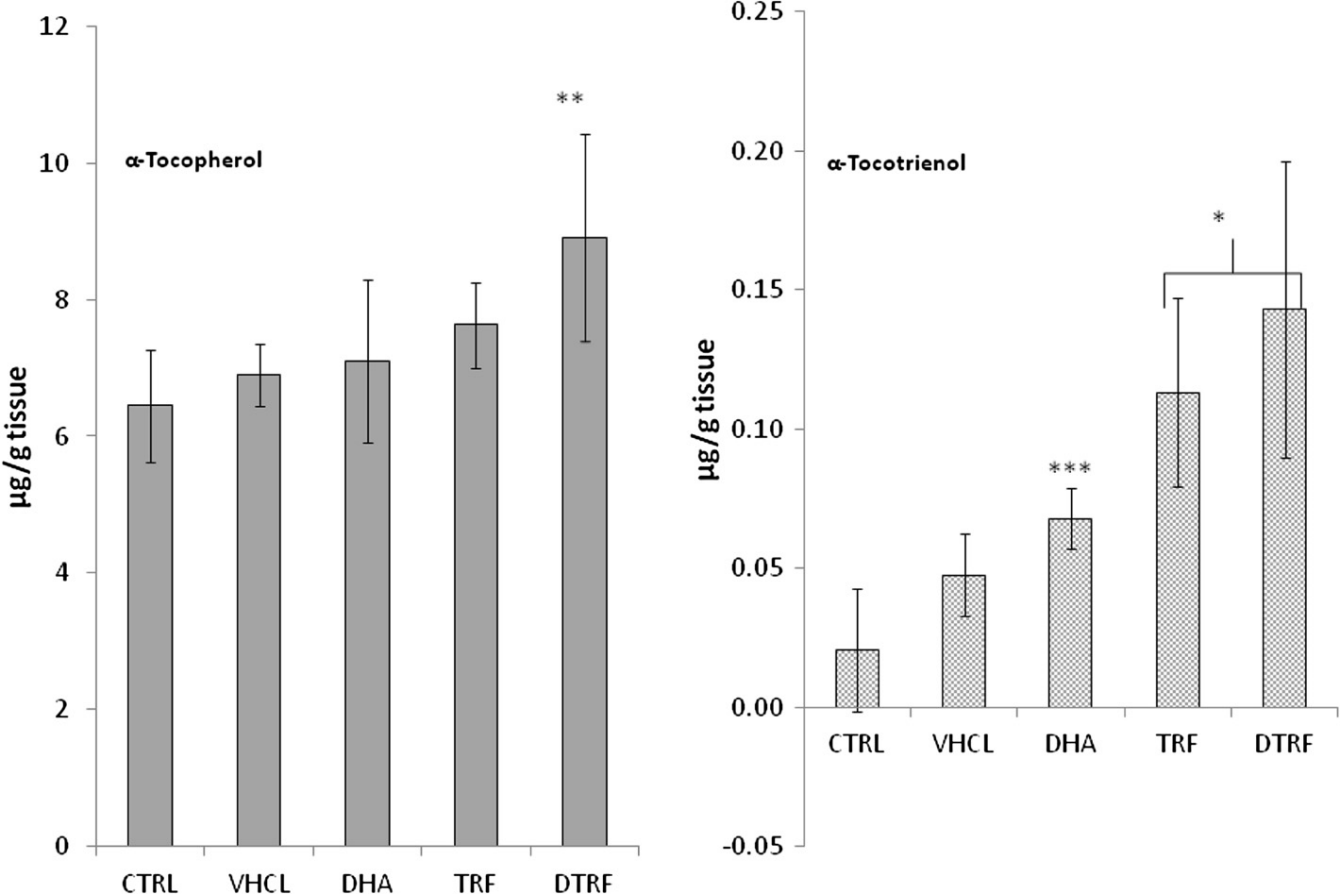
**Brain α- tocotrienol and α-tocopherols contents across treatment groups.** Values are means ± SD (n = 10). Significantly different from the control group, *P < 0.001, **P < 0.01 and ***P < 0.05.

α-Tocotrienol and α-tocopherol were the major vitamin E fractions found in the brain (Figure 2). Other vitamin E fractions were not detected. This indicated that the α-tocotrienol and α-tocopherol fractions selectively taken up by the brain tissue. α-tocopherol levels were no different in almost all groups, with the exception of an increased noted among the DTRF supplemented animals. The α-tocotrienol levels were increased among the TRF and DTRF supplemented animals.

### Effects of TRF supplementation on Morris water maze performance

#### Acquisition trial

**Figure 3:**
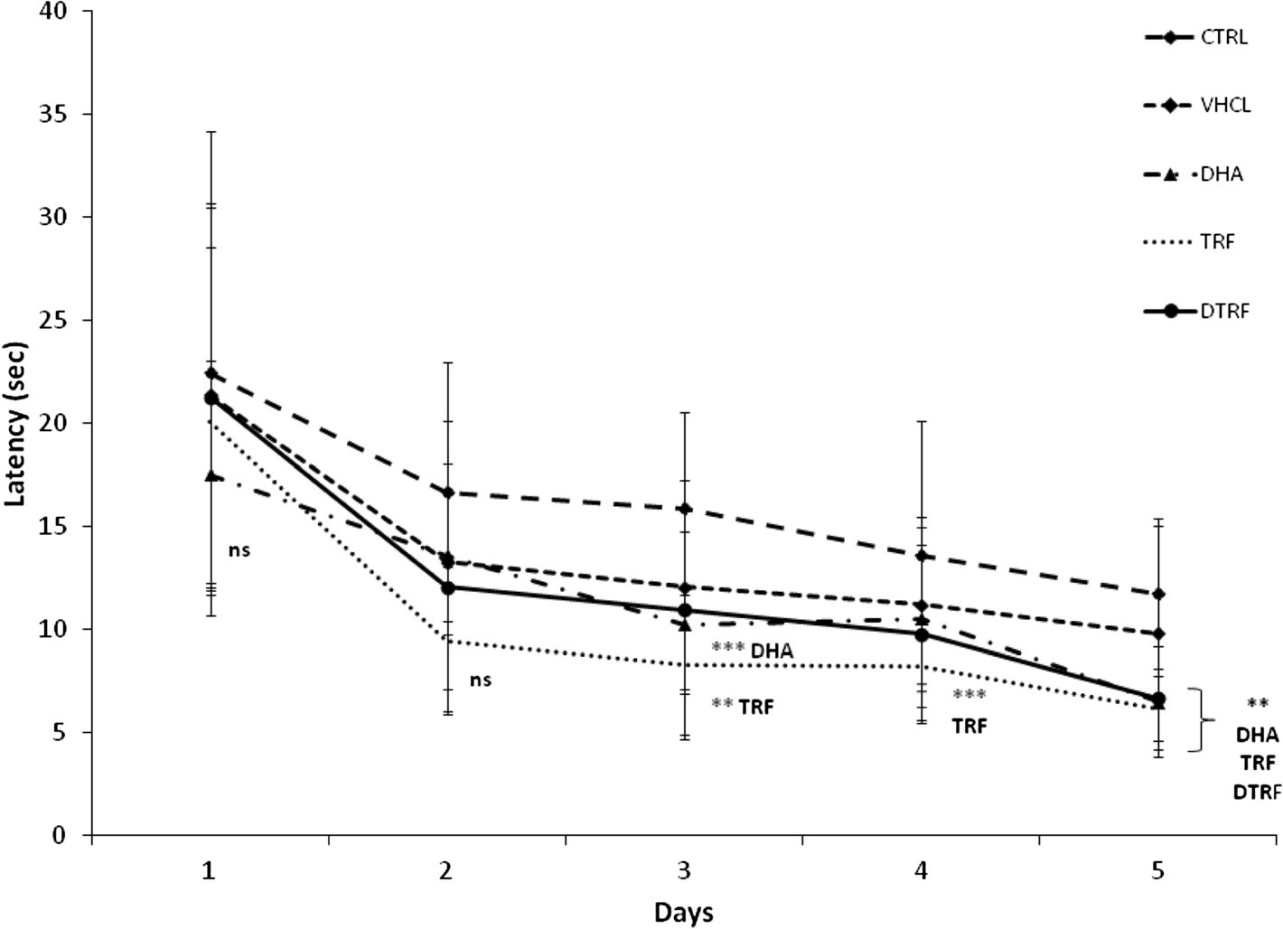
**Effects of Tocotrienol-Rich Fraction (TRF) supplementation on escape latencies across treatment groups throughout the 5 day acquisition phase.** Data expressed as mean ± SD (n = 10). **P < 0.01 and ***P < 0.05 denotes significant difference from the control group.

**Figure 4:**
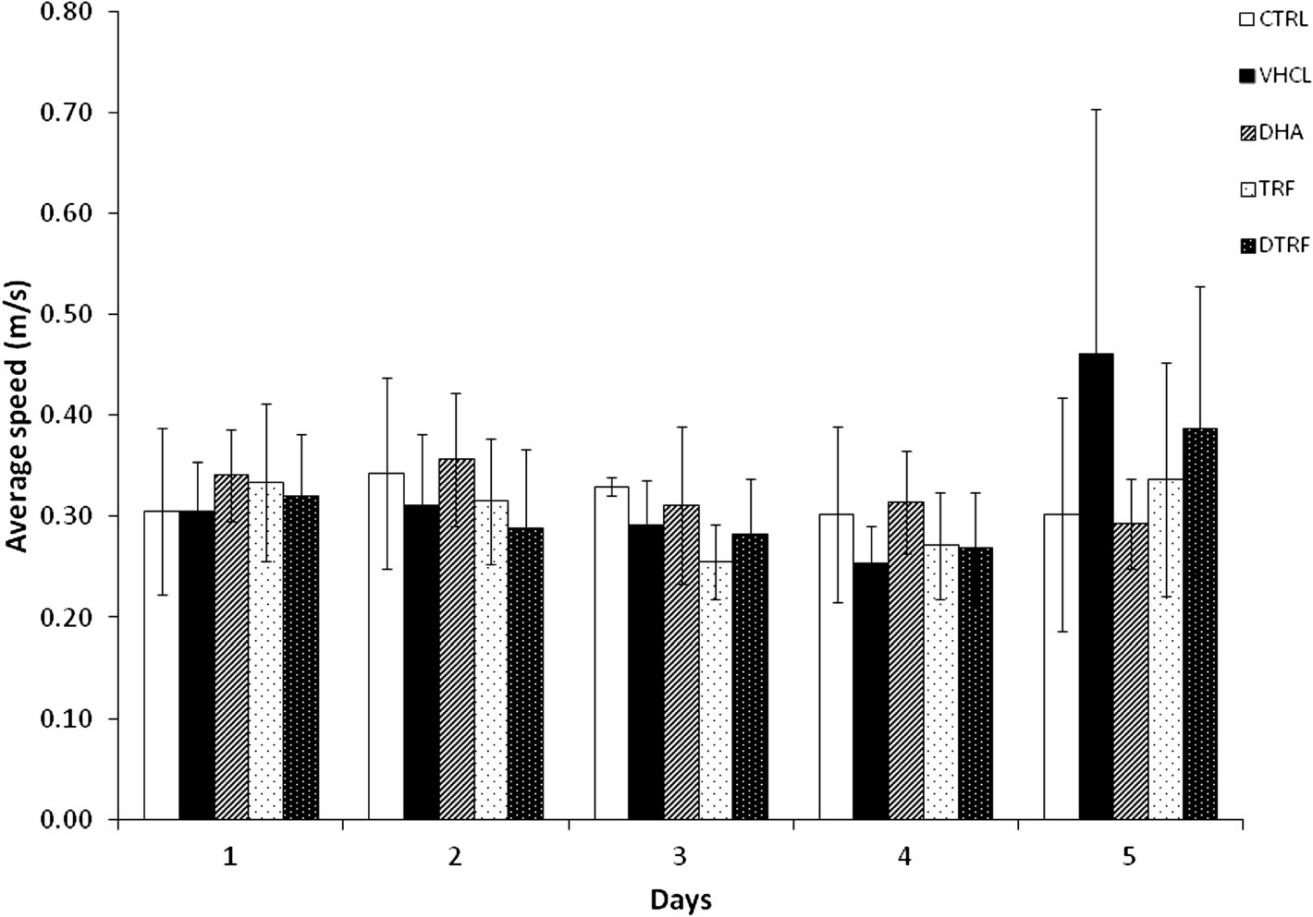
**Effects of Tocotrienol-Rich Fraction (TRF) supplementation on the average swimming speed across treatment groups throughout the 5 day acquisition phase.** Data expressed as mean ± SD (n = 10). No significant difference (P > 0.05) between treatment groups and the control group.

**Figure 5:**
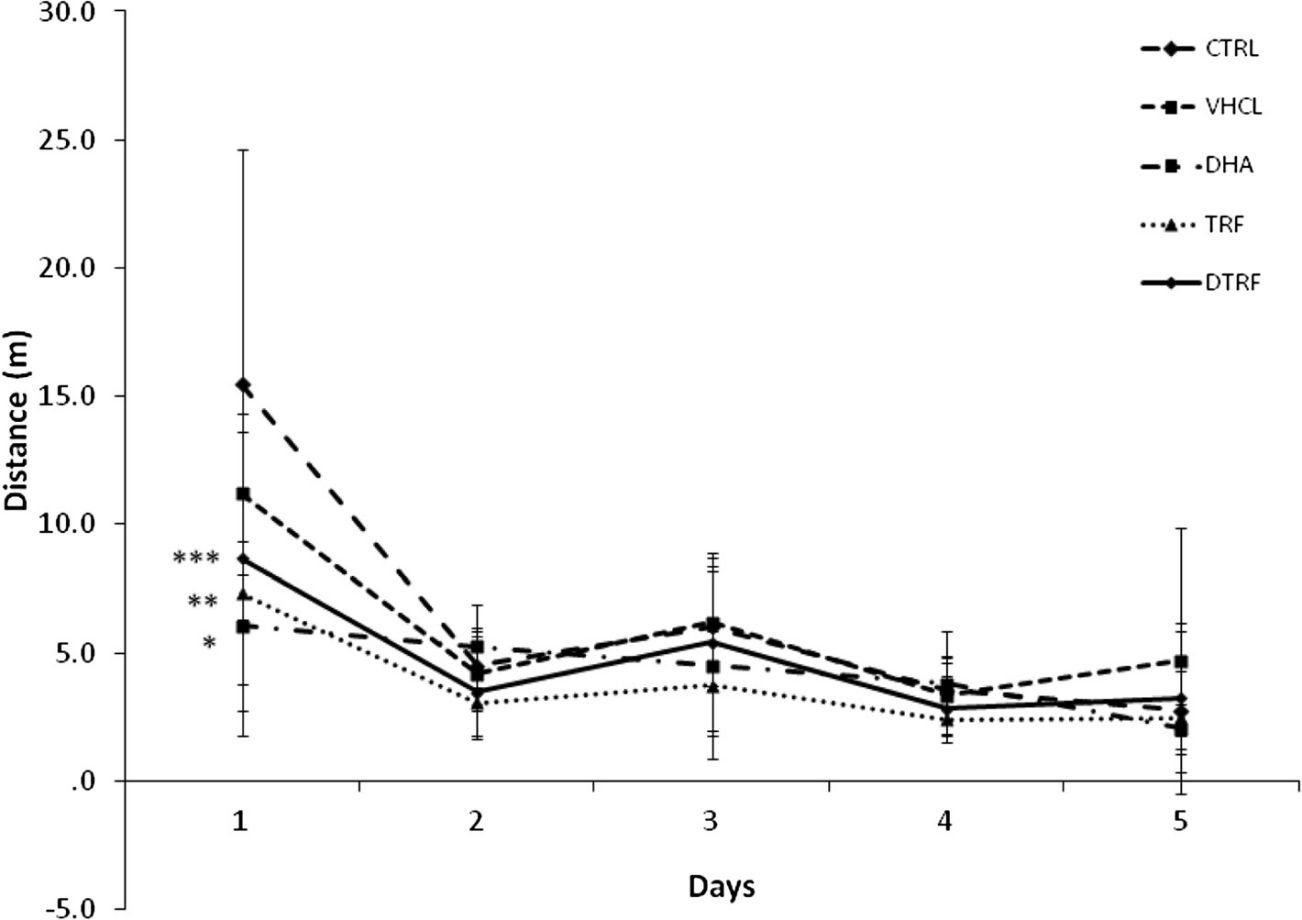
**Effects of Tocotrienol-Rich Fraction (TRF) supplementation on distance travelled across treatment groups throughout the 5 day acquisition phase.** Data expressed as mean ± SD (n = 10).* P < 0.001 **P < 0.01 and ***P < 0.05 denotes significant difference from the control group.

The effects of DHA, TRF and DTRF supplementation on spatial learning as evaluated using the Morris water maze test is depicted in Figure 3. There was a significant improvement in mean escape latency over a 5-day period (P < 0.05). This indicated that all rats learned the spatial task during the training trials. It is evident that animals supplemented with DHA and TRF demonstrated significantly better escape latency after day 3 (P < 0.05), indicating that they had learnt the position of the escape platform much more rapidly compared to the other groups after day 3. Those fed with DTRF were only able to achieve the same feat on day 5. These differences were not attributed to swimming speeds as all groups had similar swimming speeds (Figure 4). There were no differences in terms of path efficiency across groups even though path efficiency seemed to be improving with day. Similar development was also noted for distance travelled (Figure 5). It was noted that the control animals took significantly longer path (P < 0.05) to locate the platform on the first day. However, no difference was detected across groups from day 2 onwards.

**Figure 6:**
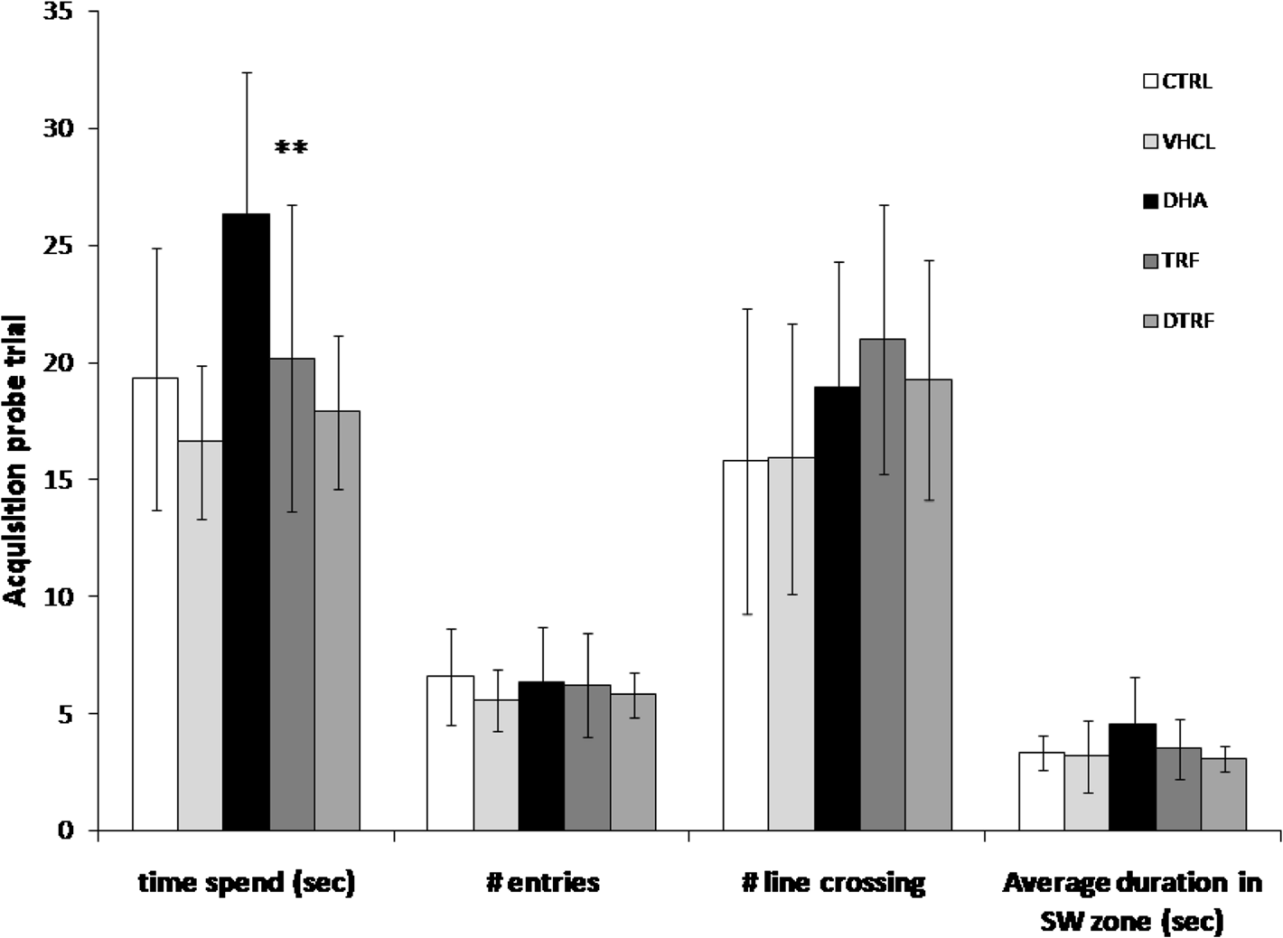
**Acquisition probe trial results across treatment groups.** Data are shown as means ± SD (n = 10). Time spent (sec), time spent in the SW quadrant, where the platform was positioned during acquisition trial; # entries, number of entries into the SW quadrant; # line crossing, number of crossing of the platform position; Average duration SW quadrant (sec), average duration spent in the SW quadrant. Significantly different from the control group, **P < 0.05.

It is interesting to note that DHA supplemented animals spent significantly more time in the quadrant where the escape platform was formerly placed in comparison to either vehicle treated or control animals (P < 0.01) during the memory retention testing procedure (Figure 6). This possibly indicates that the DHA supplemented animals remembered the prior location of the escape platform much better as compared to other groups. Other parameters or indices for probe trial such as number of entries, number of lines crossing, as well as the average duration in the area where the platform was located during training, were no different across treatment groups.

### Reversal trial

**Figure 7:**
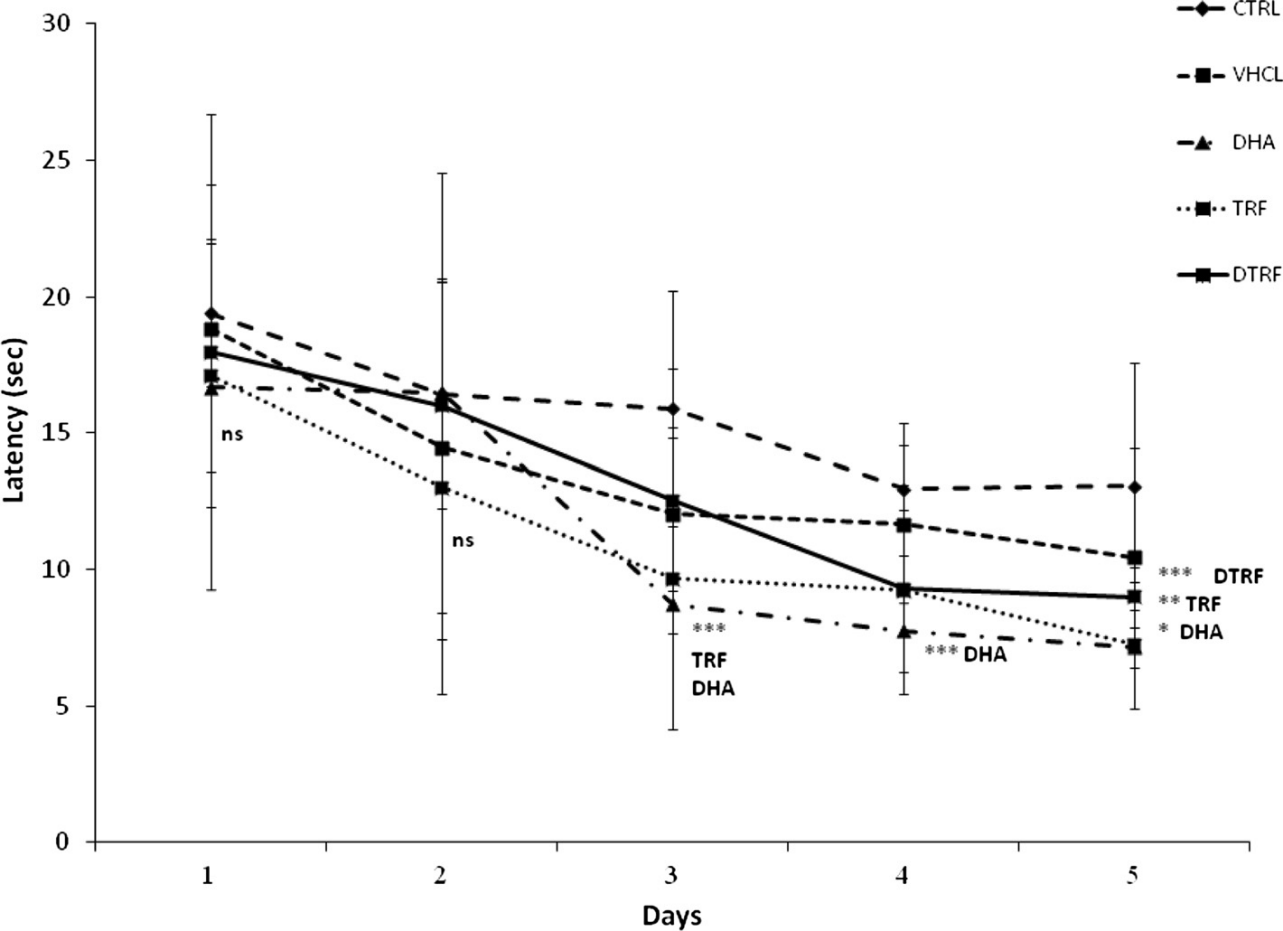
**Effects of Tocotrienol-Rich Fraction (TRF) supplementation on escape latencies across treatment groups throughout the 5 day reversal phase.** Data expressed as mean ± SD (n = 10). *P < 0.001, **P < 0.01 and ***P < 0.05 denotes significant difference from the control group.

**Figure 8:**
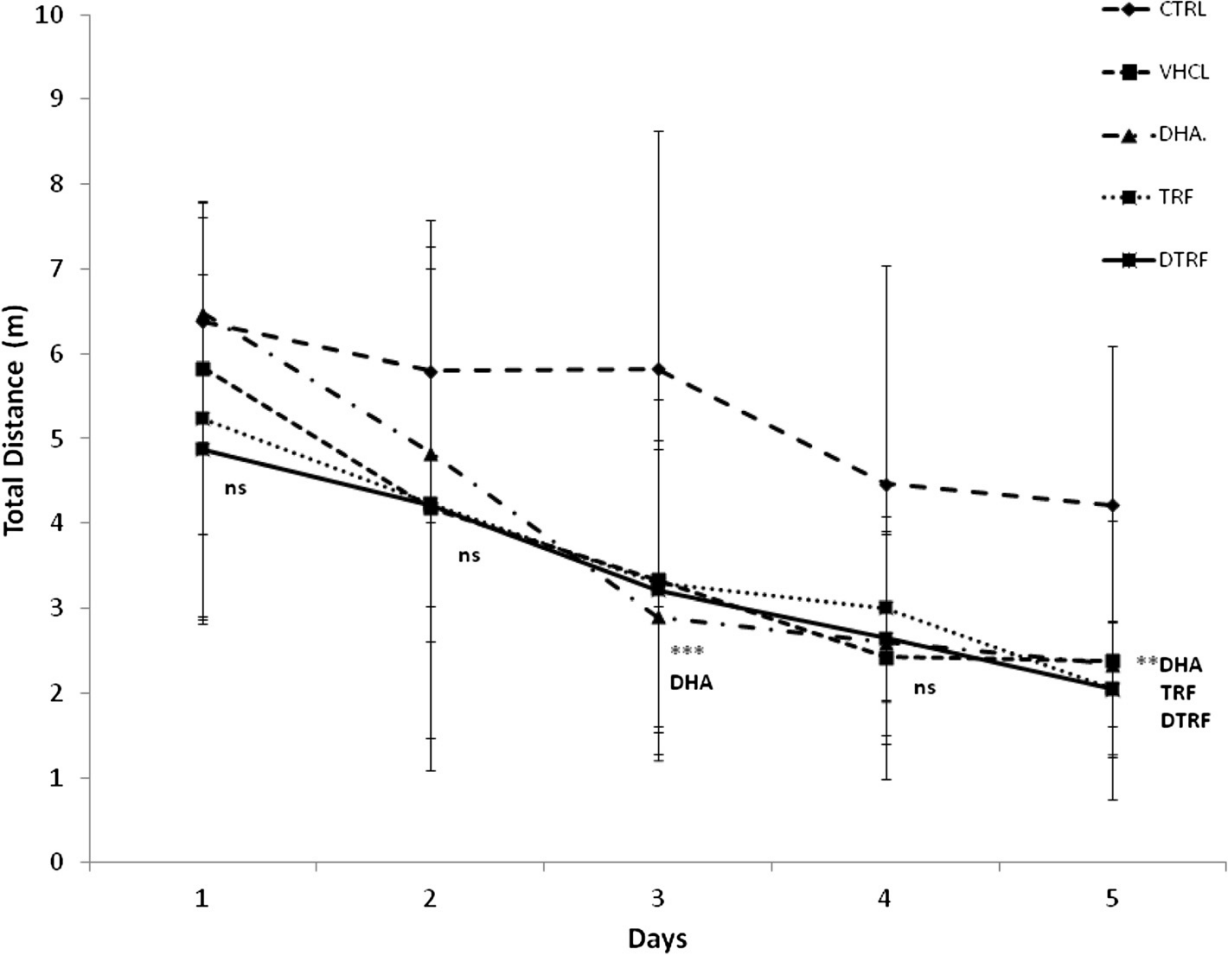
**Effects of Tocotrienol-Rich Fraction (TRF) supplementation on distance travelled across treatment groups throughout the 5 day reversal phase.** Data expressed as mean ± SD (n = 10). **P < 0.01 and ***P < 0.05 denotes significant difference from the control group.

**Figure 9:**
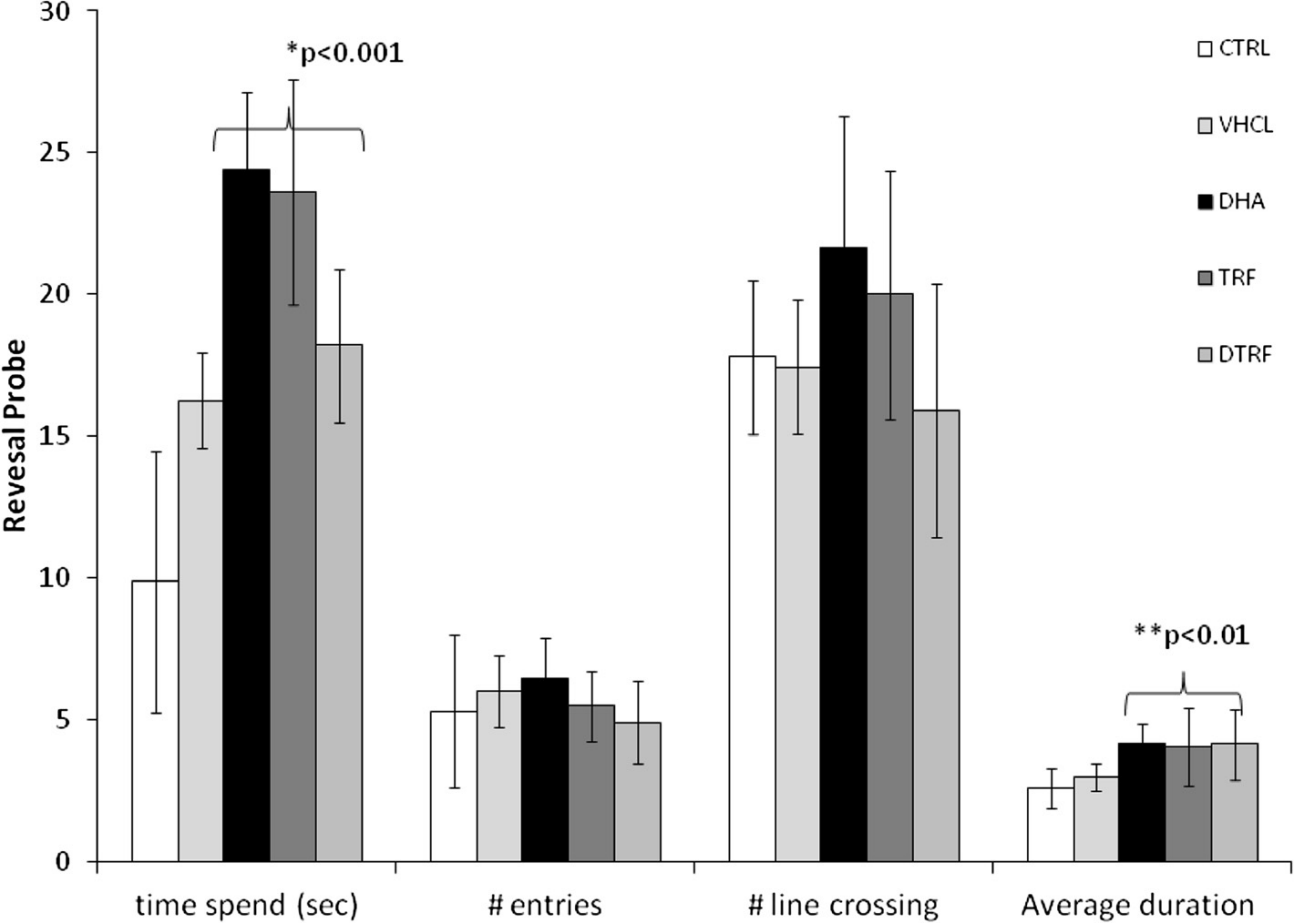
**Reverse probe trial results across treatment groups.** Data are shown as means ± SD (n = 10). Time spent (sec), time spent in the SW quadrant, where the platform was positioned during acquisition trial; # entries, number of entries into the SW quadrant; # line crossing, number of crossing of the platform position; Average duration SW quadrant (sec), average duration spent in the SW quadrant. Significantly different from the control group, *P < 0.001 and **P < 0.01.

Figure 7 depicts the results of the reversal test in which the platform was placed on the opposite side of the quadrant. The reversal test evaluated memory plasticity and re-learning abilities. Results showed that the escape latencies of all groups gradually declined over 5 days. However, significant differences between treatment groups was seen only on day 3 (P = 0. 03), day 4 (P = 0. 003) and day 5 (P < 0.001). DHA-supplemented group recorded a shorter escape latency on day 3 and day 4 (P < 0.05) compared to the control group. By day 5, in addition to the DHA group, both TRF and DTRF groups (P < 0.05) also logged a shorter escape latency compared to the control group. Collectively, these results showed that the rate of acquisition improved on day 5 in the reversal trial. The results also showed that rats from all treatment groups logged similar distances to arrive at the escape platform during the reversal trial on days 1 and 2. However, the differences were only observed on day 3 (P = 0. 02), day 4 (P = 0. 04) and day 5 (P < 0.001). TRF, DHA and DTRF supplemented animals swam significantly shorter distance compared to control animals on day 3 (P < 0.05) and day 5 (P < 0.01) (Figure 8). However, no differences were found on day 4. However swimming speeds did not differ across treatment groups. The reversal probe trial (Figure 9) also indicated that all supplemented animals had quadrant preference, all supplemented groups spent more time in NE quadrant formerly containing the platform compared to control animals.

## Discussion

The aim of the present study is to examine the possible prenatal and early postnatal effects of TRF supplementation on the cognitive function development of male progenies from dams supplemented with TRF. In general, the results demonstrated that the long-term TRF supplementation regime during the prenatal and postnatal period resulted in higher brain α-tocotrienol levels among the male off-springs. This also corresponded to significantly better cognitive performance among these rats in the Morris water maze test.

The level of tocopherol and tocotrienol enrichment in the plasma and brain of male offspring rats reflected the level of TRF supplementation among these study subjects. α-tocopherol was the major tocopherol fraction found in plasma as TTP facilitated α-tocopherol concentrations in plasma and extra-hepatic tissues [22]. The lower plasma tocotrienols level in comparison to that of tocopherols could be attributed to the lower binding affinity of tocotrienols to TTP compared to tocopherols [23]. In fact, tocotrienols have about 8.5 times less affinity to TTP compared to tocopherols [23]. Our present observation of the level of vitamin E in the brain is in line with previous studies [6,18]. In fact, it was also reported that tocotrienol uptake is higher in the fetal brain compared to the dams [17]. However, the actual role of TTP in tocotrienol transport remained to be investigated as it was not clear whether the delivery of tocotrienols to the vital organs is also TTP-dependent. It was reported that the TTP-deficient mice fed with tocopherol were infertile due to tocopherol deficiency. In contrast tocotrienol fed TTP-deficient mice were fertile, suggesting that tocotrienol was successfully delivered to the relevant organs even in the absence of TTP. Further accumulation of α-tocotrienol also observed in vital organs in TTP deficient mice supplemented with tocotrienol indicates that the tocotrienol delivery is TTP-independent [16].

The cognitive parameters of TRF supplemented animals from the current study were comparable to that of the DHA-treated group which was a positive control for this experiment. DHA is a long chain polyunsaturated fatty acids (LCPUFA) known for its beneficial effects on cognition in human infants [24] and in aged individuals [25]. The fact that all supplemented (TRF, DHA and DTRF) animals demonstrated improvement in spatial learning over a 5 day period indicates that the ability to learn improves with dietary supplementation as employed in this study. In fact our results are in agreement with a recent report on the positive effects of TRF supplementation on spatial learning and memory in 11-month old Wistar rats [21]. Previous studies have also shown that TRF supplementation prevented cognitive impairment associated with diabetes [18], chronic alcohol consumption [19] and intra-cerebroventricular streptozotocin-induced oxidative stress [20]. These studies showed that tocotrienol exerted a protective effect against oxidative damage in rat brain. Brain damage by free radicals may induce cognitive deficit via dysfunction in neurotransmission. The memory enhancing effects shown by TRF from palm oil in the present study could be at least in part, due to its antioxidant properties. Neuronal oxidative damage by reactive oxygen species (ROS) has been implicated in various neurodegenerative disorders such as Alzheimer’s disease (AD), Parkinson’s disease and dementia. Increased oxidative stress in the brain often leads to increased lipid peroxidation markers, protein oxidation, DNA and RNA damage [26]. In fact, oxidative damage to rat synapses have been proven to result in cognitive deficits [27]. Thus the protective effects of tocotrienols against peroxidative damage in the brain could be the main explanation for the improved cognitive performance seen in the current experiment. To lend further support to this fact, tocotrienols have also been known to exert a protective effect against oxidative damage in diabetes [28] and rat brain mitochondria [29], and thus the associated improvement in cognitive functions.

Our data showed shorter escape latency with TRF supplementation and these differences were not due to swimming speed as no group differences were observed in this measure. The TRF and DHA supplemented groups had equivalent swimming speed during the Morris water maze acquisition phase; we concluded that the groups were equally competent in physical ability and motor function to perform the trial. Prior to the Morris water maze test, all animals were also examined and verified to be free from physical disabilities that would affect their maze performance. Our current results also showed that TRF supplemented animals had better memory retention compared to DHA supplemented animals. This is evident in the performance of TRF supplemented animals during the probe trial. TRF supplemented animals exhibited better relearning ability in learning the new platform location during the reversal phase. During the reversal trial, it was observed that the TRF treated animals returned efficiently to the last known position of the escape platform, before starting to re-learn their way to the new location of the escape platform. This is further proof that TRF supplementation did not result in learning deficit, instead exhibiting better interpretation in the general mechanisms of acquiring, encoding or storage. These findings clearly indicate that spatial acquisition and reversal memory performances are improved with pre and post-natal TRF supplementation, possibly through mechanisms related to the improvement of hippocampal long term potentiation (LTP). The present evidence also suggests that early loads of TRF may promote long lasting learning ability in the adult progeny by influencing brain developmental process. The current study depended solely on the Morris water maze test to assess the behavioural, spatial learning and memory performance of the study subjects, similar to that reported an earlier study [30]. This is mainly because maze tests that require feed restriction such as radial arm maze would potentially result in disruption to the breeding performance of the F0 dams, and litter size for subsequent experiments employing male progenies. However, performing additional tests using other mazes would definitely add credence to the results [31].

Only male progenies were used in this study, as previous reports stated that male mice [32,33] and rats [34] performed better and yielded consistent results compared to female rats. The sex differences in memory and learning performance is known to be strongly influenced by sex hormones. Testosterone present in males has little effect on sex-differences memory and learning performance. However, the female hormone estrogen does play a role. The difference in performance between male and female was greater when females begin training in pro-oestrus cycle when oestrogen levels were high [35]. The structure of hippocampus has been shown to undergo structural changes with more dendritic spines at pro-oestrus phase when there are high levels of oestrogen. This suggests that the structural changes in the brain may rely on gonadal hormone levels which may affect the performance in behavioural tasks [36]. Besides, that oestrogen and progesterone have been shown to influence the enzymes, receptors and transporter mechanisms associated with neurotransmission, which are vital for storage and processing of memories [37]. In the light of these findings, the current study therefore placed a considerable degree of emphasis on male animals to assess spatial memory learning and performance among offsprings.

## Conclusions

In conclusion, the current study demonstrates that maternal intake of TRF increases the α-tocotrienol level in the progeny’s brain. This results in better behavioural performance and cognitive function development in the progeny. Further studies are needed to ascertain the molecular correlation between prenatal and early postnatal TRF supplementation on the synaptic function and cognition in the rat model.

## Methods

### Animals

Female Sprague Dawley rats, n = 2 per group (F0 generation, 180–200 grams, eight weeks of age), were used in this trial to breed F1 male offsprings. Animals with physical and locomotor defects that would affect their swimming ability and their performance in the Morris water maze were excluded from the trial. Only (n = 10) F1 male off-springs were included in the subsequent memory and learning trial. In total, 60 animals were used in this trial, where 10 were F0 generation female rats, and 50 F1 male rats derived from these 10 dams. All animals were kept singly in polycarbonate cages measuring (60 cm L × 45 cm W × 25 cm D), except between parturition and weaning period where each litter was housed in a cage. The animals were housed in the Animal House Facility, Faculty of Veterinary Medicine, Universiti Putra Malaysia. The animals were maintained at an ambient temperature of 24°C (± 1°C) with a 12 hour light and dark cycle. The F0 animals were acclimatized for 10 days prior to the start of the trial. Water was available *ad libitum* and animals were fed once a day at 0800 h. The experimental protocol was approved by the Animal Care and Use Committee, Faculty of Veterinary Medicine, Universiti Putra Malaysia (UPM/FPV/PS/3.2.1.551/AUP-R88).

### Treatment diets

The Ridley rat chow (Ridley Agriproducts, Sydney, Australia) was purchased from local a supplier and was used as a base diet. TRF (Gold-Tri E ™70 Batch No: GHB0903060070) with 66-70% purity was purchased from Golden Hope Bioganic Sdn. Bhd. Docosahexaenoic Acid (DHA) in the form of LONZA DHA FNO was purchased from LONZA Ltd., Switzerland and was used as positive control. The treatments were suspended in palm-based product (vehicle) which acted as a carrier to deliver the TRF and/or DHA treatment in the diet. The vehicle was added at 70 g/kg to the base diet. DHA and DTRF diets were fortified with 7 g/kg LONZA DHA FNO (containing 40-46% DHA). The nutrient compositions of the treatment diets are presented in Table 1.

**Table 1:** Nutrient composition of treatment diets

**Ingredients (base diet)**	**Amount (g/100 g diet)**				
Carbohydrate	63.3				
Crude protein	20				
Crude fat	3				
Crude fibre	7.3				
Calcium	0.9				
Phosphorus	0.5				
**Test fat**^**1**^	**CTRL**	**VHCL**	**DHA**	**TRF**	**DTRF**
Palm based product^2^	-	7.0	6.3	7.0	6.3
LONZA DHA^3^	-	-	0.7	-	0.7
Total tocopherols (mg/kg)	5.1	23.9	33.8	294.4	301.8
Total tocotrienols (mg/kg)	0.4	56.0	55.9	705.8	698.3
Total Vit E (mg/kg)	5.5	79.9	89.7	1000.2	1000.1

^1^The base diet was added with 70 g/kg of the vehicle or 63 g/kg vehicle + 7 g/kg DHA.

^2^palm based product was used as a vehicle to carry DHA and TRF.

^3^40-46% DHA by weight from Lonza Ltd.

### Experimental design

**Figure 10:**
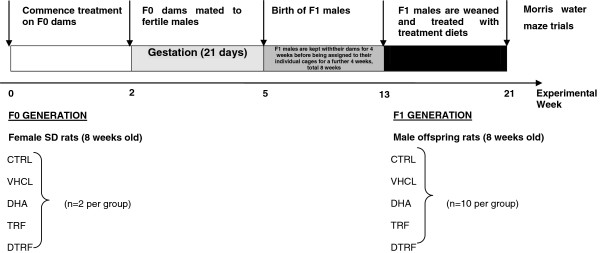
Flow diagram illustrating experimental events.

A total of 10 female rats, aged 8-week-old were used. The rats were housed individually and maintained on normal or treated rat chow. The rats were randomly assigned to five groups of two animals each (CTRL, VHCL, DHA, TRF and DTRF), and fed with the base diet as control, (CTRL), base diet plus vehicle (VHCL), base diet plus docosahexanoic acid (DHA), base diet plus Tocotrienol-Rich fraction (TRF)(100 mg/kg body weight), and base diet plus both docosahexaenoic acid and tocotrienol rich fraction (DTRF) diets for 2 weeks prior to mating. The treatments (TRF, DHA, and DTRF) were suspended in palm based product (vehicle) and laced on a base diet. Diets were prepared fresh and fed to animals once daily for 2 weeks. At 10 weeks of age, the females were mated with fertile males. The females (F0 generation) were maintained on their respective treatment diets throughout the gestation and lactation periods. Pups (F1 generation) derived from these dams were raised with their dam from birth till 4 weeks post natal. The male pups were weaned at 8 weeks postnatal, at which they were grouped into 10 animals each and maintained on the same diet as their dams for a further 8 weeks. The Morris water maze was performed on males when they were about 16 weeks old. The sequence of events explaining the experiment is illustrated in Figure 10.

### Cognitive tasks evaluation

#### Morris Water Maze test (MWM)

Testing of spatial learning in a Morris water maze was performed as described by Vorhees et al*.*[38] and [30].The maze consisted of a black plastic pool, 120 cm in diameter and 55 cm in depth, half filled with tap water 23°C (±1°C). An escape platform of 10 cm in diameter was placed in the centre of a quadrant, and submerged 2 cm below the water surface. The tank was placed in an experimental room and three spatial reference cues (shapes of circle, triangle and square) were placed around the pool. The pool was divided into four quadrants as zone NW (north-west), NE (north-east), SW (south-west) and SE (south-east). The animal’s performances were recorded by a ceiling-mounted camera (DSR-SR47; Sony Corporation, Tokyo, Japan) and analyzed with ANY-maze Video Tracking System Software (Stoelting Co., USA). Prior to the maze test, all animals were examined and verified to be free from physical disabilities and motor function deficits that would affect their maze performance. The test was carried out in three phases as described below.

### Spatial acquisition

This phase evaluated the spatial learning abilities of the rats. Improvement in spatial learning is indicated by the decreasing escape latencies. Rats had daily training for 5 consecutive days with four trials per day per treatment regime. On each trial, the rat was placed in the water, facing the edge of the pool, at one of four pseudo-randomly determined start positions. The rats were given 1 minute to locate hidden platform which was placed in the centre of SW quadrant. When the animal reached the hidden platform, it was allowed to rest for 15 seconds. If the animal failed to find the platform within 1 minute, it was physically guided to the platform.

### Probe trial

To assess the spatial memory retention, a probe trial was performed 24 hours after the last acquisition day. In this trial, the platform was removed and the animal was released from the opposite site of the quadrant and allowed to swim for 1 minute. The relative time spent in each quadrant was recorded and analyzed.

### Spatial reversal

To assess the relearning ability of the animals, reversal training, a similar task to the acquisition, was initiated 24 hours after the acquisition probe trial. Animals were trained to find a hidden platform, now relocated in the opposite quadrant (reversed) from the initial location. The trial was conducted for 5 consecutive days with 4 trials per day regime. Latency to find platform was determined in each trial. On day 6, all animals were subjected to reversal probe trial.

Dependent variables chosen for tracking during acquisition and reversal trials were: latency to the platform, total distance swam (path length), average swim speed and path efficiency (the ratio of the shortest possible path length to actual path length). All these parameters are, to a lesser or greater degree, related to goal-directed behaviour, i.e. spatial learning.

The parameters of memory, assessed during the probe trial included the time spent in the target quadrant, number of entries, number of lines crossing, as well as the average duration in the area where the platform located during training.

### Blood and tissue sampling

After the last learning trials, all the male rats were euthanized with an overdose of pentobarbital sodium (200 mg/kg) (Troy Industries Pte., Australia). 5 ml blood was collected with ethylene-diamine-tetra-acetate (EDTA) collection tubes via cardiac puncture. Blood plasma was then separated by centrifugation and stored at −80°C. The whole brains of the male rats were rapidly removed and rinsed with ice-cold phosphate buffered saline (PBS) to remove blood and blotted to remove excess water. The plasma and brain samples were stored at −80°C for vitamin E analysis.

### Extraction of vitamin E from the brain tissues

The vitamin E (tocopherols and tocotrienols) content in the brain was determined according to the methods of Patel et al*.*[39], with some modifications. 0.5 g of the brain was cut into small pieces. 1 ml of 0.9% NaCl, 1 ml of absolute ethanol and 30 μl of 2,2,5,7,8-pentamethyl-6-hydroxychroman (PMC) (internal standard, 10 ppm) were added into the sample. The mixture was then vortexed to ensure proper mixing. This was followed by homogenization for 30s in 5 ml of hexane. The mixture was vigorously shaken with (IKA- VIBRAX-VXR, Germany) shaker for 2 hours and centrifuged for 2000 rpm for, 15 minutes. The upper layer was collected and dried with nitrogen gas. The extracted samples were analysed for vitamin E content using a normal phase High Performance Liquid Chromatography (HPLC).

### Extraction of vitamin E from plasma

The vitamin E (tocopherols and tocotrienols) content in the plasma was determined according to the methods of Nesaretnam et al. [40], with some modifications. 0.3 ml of plasma was placed in a 15 ml centrifuge tube. 1 ml of 0.9% NaCl, 1 ml of absolute ethanol and 6 μl of PMC (internal standard, 10 ppm) and 5 ml of hexane were added into the sample. The mixture was vortexed to mix. The mixture was vigorously shaken with (IKA- VIBRAX-VXR, Germany) shaker for 2 hours and centrifuged at 2000 rpm for 15 minutes. The upper layer was collected and evaporated under nitrogen gas. The extracted samples were analysed for vitamin E content using normal phase High Performance Liquid Chromatography (HPLC).

### HPLC analysis

The HPLC system used for vitamin E analysis was the Agilent 1100 series HPLC machine. The HPLC is equipped with Agilent model FLD G1321A fluorescence spectrophotometer and Agilent Chemstation for LC systems Rev. A.06.0×. The mobile phase was hexane: dioxin: isopropyl alcohol (970:25:5 v/v). The degassed mobile phase was delivered at 1 ml/min flow rate. The sample was eluted on a Phenomenex®Luna 5 μM silica (250 × 4.6 mm I.D., 5 μM) column. The detector was set at an excitation wavelength of 295 nm and an emission wavelength of 325 nm. The known amount of sample was dissolved in 10 μL of eluting solvent and injected into the HPLC. The standard solution was prepared from 0.05-10 μg/ml of tocotrienol and tocopherol fractions of (α, β, δ, γ) (kind gift from Davos Life Sciences, Singapore), and 2,2,5,7,8-Pentamethyl-6-chromanol (PMC) (Sigma Aldrich, USA) was employed as the internal standard. Quantification of the major components was carried out by comparing the peak areas with those of the standards.

### Statistical analysis

Results were expressed as mean ± 1 standard deviation for all datasets. Prior to statistical analysis, all datasets were checked for their conformance to the assumption of normality. All datasets were subsequently analyzed using parameteric tests as they are normally distributed. Results for plasma alpha tocopherol and tocotrienol levels, acquisition and reversal probe trials were compared across groups using the one way analysis of variance procedure. When repeated interday comparisons are required, the repeated measures analysis of variance (RM ANOVA) method was used. Significantly different means were then elucidated using the Tukey HSD test. All statistical procedures were performed at the 95% confidence level using the PASW SPSS software version 18.0.

## Competing interests

The authors declare that they have no competing interests.

## Authors’ contributions

All authors conceived the study, participated equally in the experimental design and drafted the manuscript. All authors read and approved the final manuscript.

## References

[B1] Suzuki YJ Tsuchiya M Wassall SR Choo YM Govil G Kagan VE Packer L Structural and dynamic membrane properties of. Alpha.-tocopherol and. Alpha.-tocotrienol: implication to the molecular mechanism of their antioxidant potency Biochemistry 1993 32 10692 11069 10.1021/bi00091a020 8399214

[B2] Azlina MFN Nafeera MI Khalid BAK Effects of tocotrienol on lipid peroxidation in experimental gastritis induced by restraint stress Pakistan J Nutr 2005 4 2 69 72

[B3] Das S Lekli I Das M Szabo G Cardioprotection with palm tocotrienols: comparisons with different isomers Am J Physiol Heart Cirl Physiol 2008 294 2 70 78 10.1152/ajpheart.01200.2007 18083895

[B4] Qureshi AA Qureshi N Wright JJK Shen Z Kramer G Gapor A Chong YH Dewitt G Ong A Peterson DM Lowering of serum cholesterol in hypercholesterolemic humans by tocotrienols (palmvitee) Am J Clin Nutr 1991 53 1021S 1026S 2012010 10.1093/ajcn/53.4.1021S

[B5] Nesaretnam K Ambra R Selvaduray KR Radhakrishnan A Reimann K Razak G Virgili F Tocotrienol-rich fraction from palm oil affects gene expression in tumors resulting from MCF-7 cell inoculation in athymic mice Lipids 2004 39 5 459 467 10.1007/s11745-004-1251-1 15506241

[B6] Khanna S Roy S Slivka A Craft KST Chaki S Rink C Notestine AM Devries C Parinandi NL Sen CK Neuroprotective properties of the natural vitamin E α-tocotrienol Stroke 2005 36 e144 e152 10.1161/01.STR.0000181082.70763.22 PMC1829173 16166580

[B7] Park HA Kubicki N Gnyawali S Chan YC Roy S Khanna S Sen CK Natural vitamin E α-tocotrienol protects against ischemic stroke by induction of multidrug resistance-associated protein 1 Stroke 2011 42 8 2308 2314 10.1161/STROKEAHA.110.608547 21719775 PMC3362046

[B8] Wainwright PE Colombo J Nutrition and the development of cognitive functions: interpretation of behavioral studies in animals and human infants Am J Clin Nutr 2006 84 5 961 970 17093144 10.1093/ajcn/84.5.961

[B9] Dauncey MJ Bicknell RJ Nutrition and neurodevelopment: mechanisms of developmental dysfunction and disease in later life Nutr Res Rev 1999 12 2 231 253 10.1079/095442299108728947 19087453

[B10] Gomez-Pinilla F Brain foods: the effects of nutrients on brain function Nat Rev Neurosci 2008 9 568 578 18568016 10.1038/nrn2421 PMC2805706

[B11] Molteni R Barnard RJ Ying Z Roberts CK Gomez-Pinilla F A high-fat, refined sugar diet reduces hippocampal brain-derived neurotrophic factor, neuronal plasticity, and learning Neurosci 2002 112 4 803 814 10.1016/S0306-4522(02)00123-9 12088740

[B12] Rassin DK Smith KE Nutritional approaches to improve cognitive development during infancy: antioxidant compounds Acta Paediatr 2003 92 34 41 12948003 10.1111/j.1651-2227.2003.tb00661.x

[B13] Lucas A Role of nutritional programming in determining adultmorbidity Arch Dis Child 2001 71 4 288 290 7979518 10.1136/adc.71.4.288 PMC1030003

[B14] Lucas A Morley R Isaacs E Nutrition and mental development Nut Rev 2001 59 8 S24 S33 10.1111/j.1753-4887.2001.tb05499.x 11519666

[B15] Lucas A Programming by early nutrition: an experimental approach J Nutr 1998 128 401S 406S 9478036 10.1093/jn/128.2.401S

[B16] Khanna S Patel V Rink C Roy S Sen CK Delivery of orally supplemented α-tocotrienol to vital organs of rats and tocopherol-transport protein deficient mice Free Radical Bio Med 2005 39 10 1310 1319 10.1016/j.freeradbiomed.2005.06.013 16257640 PMC1820629

[B17] Roy S Lado BH Khanna S Sen CK Vitamin E sensitive genes in the developing rat fetal brain: a high-density oligonucleotide microarray analysis FEBS Lett 2002 530 1–3 17 23 12387859 10.1016/s0014-5793(02)03309-4 PMC1832147

[B18] Kuhad A Bishnoi M Tiwari V Bishnoi M Chopra K Suppression of NF-κß signalling pathway by tocotrienol can prevent diabetes associated cognitive deficits Pharmacol Biochem Behav 2009 92 2 251 259 10.1016/j.pbb.2008.12.012 19138703

[B19] Tiwari V Kuhad A Chopra K Suppression of neuro-inflammatory signaling cascade by tocotrienol can prevent chronic alcohol-induced cognitive dysfunction in rats Behav Brain Res 2009 203 2 296 303 10.1016/j.bbr.2009.05.016 19464322

[B20] Tiwari V Kuhad A Bishnoi M Chopra K Chronic treatment with tocotrienol, an isoform of vitamin E, prevents intra cerebroventricular streptozotocin-induced cognitive impairment and oxidative-nitrosative stress in rats Pharmacol Biochem Behav 2009 93 2 183 189 10.1016/j.pbb.2009.05.009 19464315

[B21] Taridi NM Yahaya MF Teoh SL Latiff AA Ngah WZ Das S Mazlan M Tocotrienol rich fraction (TRF) supplementation protects against oxidative DNA damage and improves cognitive functions in Wistar rats Clin Ter 2011 162 2 93 98 21533313

[B22] Traber MG Vitamin E nuclear receptors and xenobiotic metabolism Arch Biochem Biophys 2004 423 1 6 11 10.1016/j.abb.2003.10.009 14989258

[B23] Hosomi A Arita M Sato Y Kiyose C Ueda T Igarashi O Arai H Inoue K Affinity for [alpha]-tocopherol transfer protein as a determinant of the biological activities of vitamin E analogs FEBS Lett 1997 409 1 105 108 10.1016/S0014-5793(97)00499-7 9199513

[B24] Makrides M Robert AG McPhee AJ Yelland L Quinlivan J Ryan P Effect of DHA supplementation during pregnancy on maternal depression and neurodevelopment of young children: a randomized controlled trial JAMA 2010 304 15 1675 1683 10.1001/jama.2010.1507 20959577

[B25] Yurko-Mauro K McCarthy D Rom D Nelson EB Ryan AS Blackwell A Salem N Jr Stedman M Beneficial effects of docosahexaenoic acid on cognition in age-related cognitive decline Alzheimers Dement 2010 6 6 456 464 10.1016/j.jalz.2010.01.013 20434961

[B26] Halliwell B Oxidative stress and neurodegeneration: where are we now? J Neurochem 2006 97 6 1634 1658 10.1111/j.1471-4159.2006.03907.x 16805774

[B27] Tuzcu M Giyasettin B Effect of melatonin and vitamin E on diabetes-induced learning and memory impairment in rats Eur J Pharmaco 2006 537 1 106 110 10.1016/j.ejphar.2006.03.024 16626697

[B28] Kanaya Y Doi T Sasaki H Fujita A Matsuno S Okamoto K Nakano Y Rice bran extract prevents the elevation of plasma peroxylipid in KKAy diabetic mice Diabetes Res Clin Pr 2004 66 S157 S160 10.1016/j.diabres.2003.09.021 15563968

[B29] Kamat JP Devasagayam TPA Tocotrienols from palm oil as potent inhibitors of lipid peroxidation and protein oxidation in rat brain mitochondria Neurosci Lett 1995 195 3 179 182 10.1016/0304-3940(95)11812-B 8584204

[B30] Hajar T Goh YM Rajion MA Vidyadaran MA Othman F Tan AI Ebrahimi M Omega 3 polyunsaturated fatty acid improves spatial learning and hippocampal Peroxisome Proliferator Activated Receptors (PPARalpha and PPARgamma) gene expression in rats BMC Neurosci 2012 13 1 109 216 10.1186/1471-2202-13-109 22989138 PMC3465241

[B31] Hodges H Maze procedures: the radial-arm and water maze compared Cognitive Brain Res 1995 3 3 167 181 10.1016/0926-6410(96)00004-3 8806020

[B32] Berger-Sweeney J Arnold A Gabeau D Mills J Sex differences in learning and memory in mice: effects of sequence of testing and cholinergic blockade Behav Neurosci 1995 109 5 859 873 8554711 10.1037//0735-7044.109.5.859

[B33] Lamberty Y Gower AJ Investigation into sex-related differences in locomotor activity, place learning and passive avoidance responding in NMRI mice Physiol Behav 1988 44 6 787 790 10.1016/0031-9384(88)90063-7 3249753

[B34] Perrot-Sinal TS Kostenuik MA Ossenkopp KP Kavaliers M Sex differences in performance in the Morris water maze and the effects of initial nonstationary hidden platform training Behav Neurosci 1996 110 6 1309 1320 8986334 10.1037//0735-7044.110.6.1309

[B35] Dalla C Edgecomb C Whetstone AS Shores TJ Females do not express learned helplessness like males do Neuropsychopharmacology 2008 33 7 1559 1569 10.1038/sj.npp.1301533 17712351

[B36] Sutcliffe JS Marshall KM Neill JC Influence of gender on working and spatial memory in the novel object recognition task in the rat Behav Brain Res 2007 177 1 117 125 10.1016/j.bbr.2006.10.029 17123641

[B37] Williams CL Barnett AM Meck WH Organisational effects of early gonadal secretions on sexual differentiation in spatial memory Behav Neurosci 1990 104 1 84 97 2317288 10.1037//0735-7044.104.1.84

[B38] Vorhees CV Williams MT Morris water maze: procedures for assessing spatial and related forms of learning and memory Nat Protoc 2006 1 848 858 10.1038/nprot.2006.116 17406317 PMC2895266

[B39] Patel V Khanna S Roy S Ezziddin O Sen CK Natural vitamin E alpha-tocotrienol: retention in vital organs in response to long-term oral supplementation and withdrawal Free Radic Res 2006 40 763 771 16984003 10.1080/10715760600672491

[B40] Nesaretnam K Mahalingam D Radhakrishnan AK Premier R Supplementation of tocotrienol‒rich fraction increases interferon‒gamma production in ovalbumin‒immunized mice Eur J Lipid Sci Tech 2010 112 5 531 536

